# Assessing the Feasibility of an Intensified Extended Contact Survey (IECS) Compared to Passive Household Screening for Leprosy in Bangladesh

**DOI:** 10.3390/tropicalmed10100274

**Published:** 2025-09-24

**Authors:** Kazi Naher, Mahfuza Rifat, Dipak Kumar Biswas, Sheikh Mohammad Faisal, Nimer Ortuño-Gutiérrez, Epco Hasker

**Affiliations:** 1 Damien Foundation, H10 Rd 96, Dhaka 1212, Bangladesh; mariam@damienfoundation-bd.com (K.N.); dipak@damienfoundation-bd.com (D.K.B.); faisal@damienfoundation-bd.com (S.M.F.); 2 Damien Foundation, Boulevard Léopold II 263, 1081 Brussels, Belgium; ortunonimer@gmail.com; 3 Institute of Tropical Medicine, Nationalestraat 155, 2000 Antwerp, Belgium; ehasker@itg.be

**Keywords:** active case detection, mapping, leprosy, neglected tropical disease

## Abstract

Bangladesh is among the 23 WHO priority countries for leprosy, with a new case detection rate of 21 per million population including children among new cases indicating recent transmission. We aimed to compare active versus passive case detection using geospatial tools. A cluster-randomized study was conducted across seven intervention and seven control districts. In the intervention arm, GPS coordinates of new cases were recorded, and contacts within a 75-m buffer were screened. Spatial cluster analysis using Kulldorff’s scan statistics was performed to identify hotspots. The main objective was to achieve early case detection in intervention areas, reflected in a lower proportion of new patients with grade 2 disabilities (G2Ds). A total of 347/382 (90%) index cases were enrolled in the intervention arm, compared to 380/462 (82%) in the control arm. Among household contacts, 7/1482 (5‰) new cases were found in the intervention area and 12/1565 (7.7‰) in the control area. Additionally, 18/25,720 (0.7‰) new cases were detected among neighbor contacts in the intervention arm. G2D proportions were not lower in the intervention arm (15%) than in the comparator arm (11%, *p* = 0.043). Comparable household contact cases were observed in both arms, with more cases emerging among neighbors in intervention districts. Eight spatial clusters were identified, including 288/844 (34%) index cases, with three significant clusters from 2022 to 2023. Screening within a 75-m buffer detected additional leprosy cases, though fewer than expected due to COVID-19 limitations. Targeting clusters for case detection and prophylaxis could strengthen transmission control efforts.

## 1. Introduction

Leprosy is an ancient chronic infectious disease caused mainly by *Mycobacterium leprae*, affecting the skin, peripheral nerves, respiratory mucosa, and eyes. Leprosy has been included by the World Health Organization (WHO) as one of the 21 neglected tropical diseases (NTDs) [1,2]. Early treatment of leprosy with multidrug therapy (MDT) and prevention and management of immunological reactions can prevent permanent disability. Nevertheless, those afflicted with visible deformity often endure stigmatization and discrimination. Worldwide, for years, the number of new cases has plateaued up to 200,000 annually. In 2023, there were 182,815 new cases reported globally, of which 72% were detected in Southeast Asia (mainly India and Indonesia) [3].

Bangladesh is one of the 23 priority countries for leprosy control according to the WHO [1]. In 2023, Bangladesh reported 3639 new cases, equivalent to a new case detection rate of 21 per million inhabitants [3]. Since 2013, nationwide, the average annual number of new cases has been 3448, with the proportion of grade 2 disabilities (G2Ds), which is the presence of visible deformities of hands and/or feet and/or damage to the eyes resulting in a visual acuity below 0.1, at 8% in adults and 6% in children. However, G2Ds could be as high as ≥50% in the referral hospitals, illustrating challenges in access to early care [4]. Leprosy transmission remains stable nationwide, with some districts bearing a high proportion of children among new cases in hotspots of the Northwest, indicating ongoing transmission [5]. This fact illustrates the need to implement new interventions to enhance early diagnosis, reaching the target of the absence of children among new leprosy patients detected over five years [6].

In high endemic settings in Bangladesh, 25% of close contacts had a family history of leprosy compared to 62% in low endemic areas, implying that in high endemic settings, a wider circle of contacts needs to be screened to cut transmission [7]. In similar high endemic settings for leprosy, it has been documented that clustering occurs beyond households of the index cases, supporting the rationale for screening contacts beyond the household [8,9,10,11,12]. It is also known that active case finding (ACF) activities reduce leprosy transmission by identifying individuals with active or subclinical infections, accelerating diagnosis and treatment, preventing disability, and stopping transmission in highly endemic regions [13]. However, a cost-effective approach to active case finding (ACF) in the Bangladesh context remains to be identified.

In Bangladesh, routine leprosy services are mainly provided through passive case finding activities augmented by screening of household contacts of new leprosy patients detected attending the health facilities. In this study, we describe an active case detection approach based on door-to-door screening for leprosy of contacts residing within a buffer of 75 m surrounding a household of an index case and compare it to screening household contacts only. The 75-m buffer was chosen based on evidence of transmission risk and operational feasibility as there is a high population density in Bangladesh. Thus, we aim to achieve early case detection and decrease the proportion of new patients presenting with G2Ds in the intervention arm with ACF. We also wanted to assess geospatial clustering in both intervention and control areas at the subdistrict level in order to adapt the case detection activities in the future. Our findings will guide interventions in high-transmission areas in Bangladesh to reach the elimination of transmission of *M. leprae*.

## 2. Materials and Methods

### 2.1. Study Setting

Apart from being a high-priority country for leprosy, Bangladesh, in South Asia, is also one of the most populated countries, with an estimated 171 million inhabitants. In 2023, the Human Development Index (HDI) in Bangladesh was 0.670, and life expectancy at birth was 73.7 years, ranked at the 129th position as a medium human developed country among 193 countries [14]. 

Bangladesh has 64 districts, of which 26 are covered by nine non-governmental organizations (NGOs) for leprosy control. The Damien Foundation, a Belgian NGO, has been supporting the National Leprosy Elimination Program (NLEP) since 1972, covering 14 districts (Faridpur, Rajbari, Madaripur, Shariatpur, Gopalganj, Tangail, Mymensingh, Kishoreganj, Netrakona, Jamalpur, Sherpur, Naogaon, Nawabganj, and Rajshahi), accounting for around 33 million population.

### 2.2. Study Sites

In this study 14 districts supported by the Damien Foundation were randomly allocated to intervention or control arms after being grouped in pairs according to the average G2D rate of a preceding period of five years (2013–2017). All newly registered leprosy patients detected during the study period were eligible for inclusion. The only difference between intervention and non-intervention districts was in the way contacts were selected; i.e., screening was extended to neighborhood contacts residing within a 75-m radius around the households of index cases in intervention areas. Care for leprosy patients remained exactly the same. In accordance with current practice, clinic staff continued to use a contact register in which they recorded the names (with relationship) of family members of each new leprosy patient, date of visit, and result of examination. Patients who did not consent to participate in the study were offered the current standard of care according to the National Leprosy Guidelines.

### 2.3. Study Design

As explained above, this was a cluster-randomized intervention trial that took place from March 2020 to December 2023 in 14 districts of Bangladesh supported by the Damien Foundation. After being ranked into 7 pairs according to decreasing proportion of G2Ds among new patients detected in the preceding 5-year period, within each pair one district was randomly allocated to intervention, the other to control. The control districts consisted of Faridpur, Gopalganj, Shariatpur, Nawabganj, Rajshahi, Mymensingh, and Kishorganj and the intervention districts included Naogaon, Netrokona, Tangail, Jamalpur, Sherpur, Madaripur, and Rajbari.

In the seven intervention districts, consenting index leprosy patients were provided with a Global Positioning System (GPS) logger at the time of a monthly visit to the clinic to collect their multidrug therapy (MDT) for leprosy. The GPS logger was kept at the home of the patients and returned during the next visit. It was set to record a GPS point every three minutes during the first week. This allowed an easy identification of the exact location of the home of the index case by plotting the coordinates on a map using QGIS software (version 3.1). A 75-m radius was then drawn around the household of the index case for conducting an intensive extended contact survey (IECS) through door-to-door visits [15]. New leprosy patients in 14 districts who were diagnosed during the study period and household and neighborhood contacts who agreed to participate in screening were included in the study. People who are not permanent residents were examined if they desired but were not included in the study.

Data on all individuals screened were recorded in Open Data Kit Collect (ODK), an Android app (version v1.22.4). In control areas, the leprosy screening among contacts was limited to household members of index cases who were invited to come to their respective health facilities.

### 2.4. Sample Size

Over the period 2013–2017 a total of 1950 new leprosy cases were diagnosed in the districts supported by DFBD, i.e., 390 per year on average. Out of those, 277 (14%) presented with G2Ds. Assuming an expected 7% G2Ds at diagnosis in the intervention group and 14% in the control group, with an α level of 5% and a power (1 − β) of 80%, a minimum of 328 newly detected leprosy patients in each group was required for this difference to become statistically significant.

### 2.5. Data Collection

In the seven intervention districts, new leprosy patients and their contacts were informed about the IECS strategy and requested for written informed consent. Consenting patients were provided a GPS logger to record the geographic coordinates of their households. Thus, we were able to outline a buffer zone around each index case household, wide enough to minimize community stigma and discrimination for the index case and facilitate screening of neighbors within the study perimeter. Screening focused on skin conditions and leprosy symptoms. Screening staff included Medical Officers, Monitoring and Evaluation Officers, Tuberculosis and Leprosy Control Officers (TLCOs), and Tuberculosis and Leprosy Control Assistants (TLCAs). Contact screening required verbal informed consent, with referrals to Primary Health Care services for non-leprosy conditions. Presumptive leprosy cases were assessed on-site by a TLCO, and confirmed cases were referred to the Damien Foundation Hospital or Damien Foundation-supported subdistrict-level government health facilities (Upazilla Health Complexes) for management.

In the seven control districts, the leprosy screening among contacts was limited to household members of index cases only. Their consent was taken for participation in this study. A routine data collection and a questionnaire on patient delay were conducted. The data of the patient and their household members were collected on Android phones.

**Figure 1:**
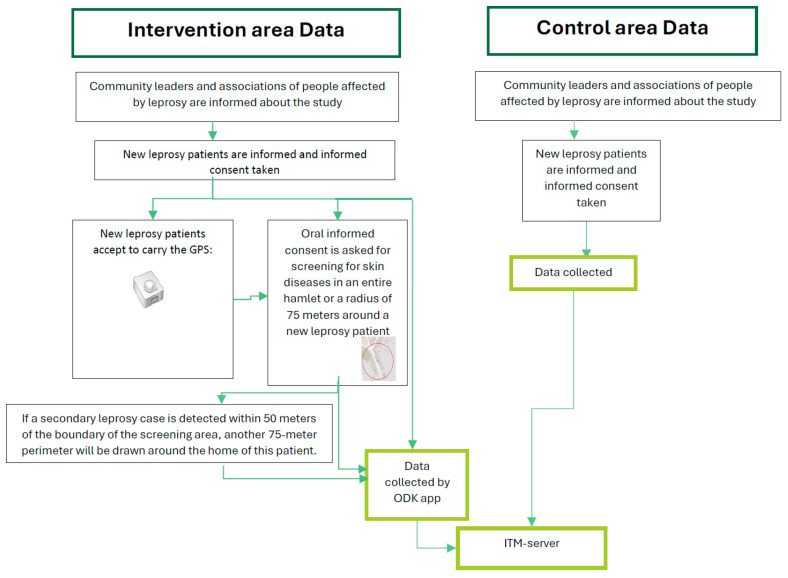
Procedures of data collection in the intervention and control arms, Bangladesh, 2020–2023. ODK = Open Data Kit; app = application; ITM = Institute of Tropical Medicine.

Patient data from both intervention and control areas were collected, verified, and uploaded to the secure ITM server using a standardized process managed through ODK software (Figure 1). The data collected in the application were cross-checked with the available leprosy program tools, the leprosy treatment card and register, in both arms.

### 2.6. Data Analysis

Data analysis was performed in STATA Version 15 (Stata Corp, College Station, TX, USA) to assess numbers and proportions of cases detected per person screened, the proportion of G2Ds among new leprosy cases, and the proportion of children among new cases. For comparison of proportion of frequencies, we used the *p*-value, for which ≤0.05 was considered statistically significant.

We used QGIS Version 3.1 to draw a 75-m perimeter around index cases. GPX files of the buffers were exported to the GPX-viewer Android app (version 1.4), allowing field staff to determine their exact position in relation to the buffer zone outlined during the screening of contacts to ensure that households included in the buffer were screened.

The spatial cluster analysis was performed to assess clustering at the subdistrict level using SaTScan Version 10.1, with Kulldorff’s scan statistic, using the discrete Poisson model, with cluster size set to a maximum of 10% of the population at risk for the identification of clusters of high transmission [16]. We used population estimates and shape files from the Humanitarian Data Exchange (HDX), which is an open-source platform for data sharing across crises and organizations managed by the United Nations Office for the Coordination of Humanitarian Affairs (UNOCHA) [17].

## 3. Results

**Figure 2:**
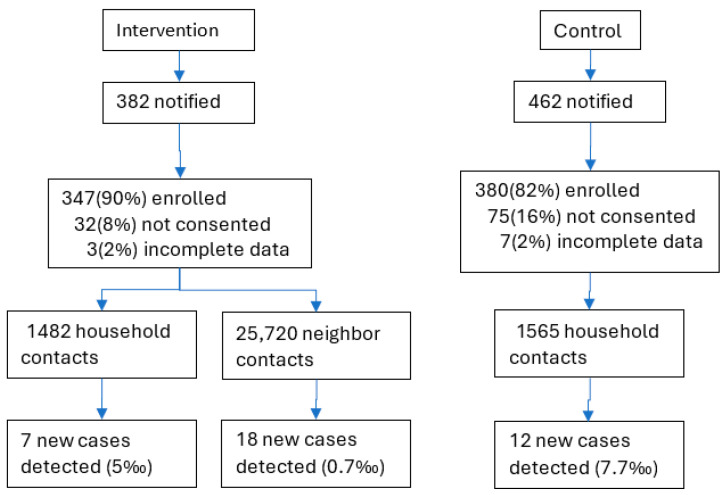
Enrollment of index cases and contacts per intervention and control arm, Bangladesh, 2020–2023.

A total of 382 and 462 leprosy patients were notified in the intervention and control arms, respectively, during the study period. About 16% of patients refused participation in the control arm, and 8%, refused participation in the intervention arm. In the control area, 1565 household contacts were screened against the index cases, which yielded 12 new cases. In the intervention arm, 1482 household contacts and 25,720 neighborhood contacts were screened, which yielded 7 new cases amongst the household contacts and 18 new cases amongst the neighborhood contacts (Figure 2).

The proportion of new patients with grade 2 disability was higher in the intervention arm (15.2%) compared to the control arm (11.1%), with a *p*-value of 0.043.

Table 1 below shows the sociodemographic and clinical characteristics of the enrolled index cases in both arms. In the control arm, 58% were males as opposed to 57% males in the intervention arm. The numbers of people enrolled under 15 years were similar: 9.5% in the control arm and 9.8% in the intervention arm. The multibacillary (MB) proportion was 56% in the intervention arm compared to 48% in the control arm.

Table 2 describes the sociodemographic data and clinical characteristics of the newly detected cases in both arms.

**Figure 3:**
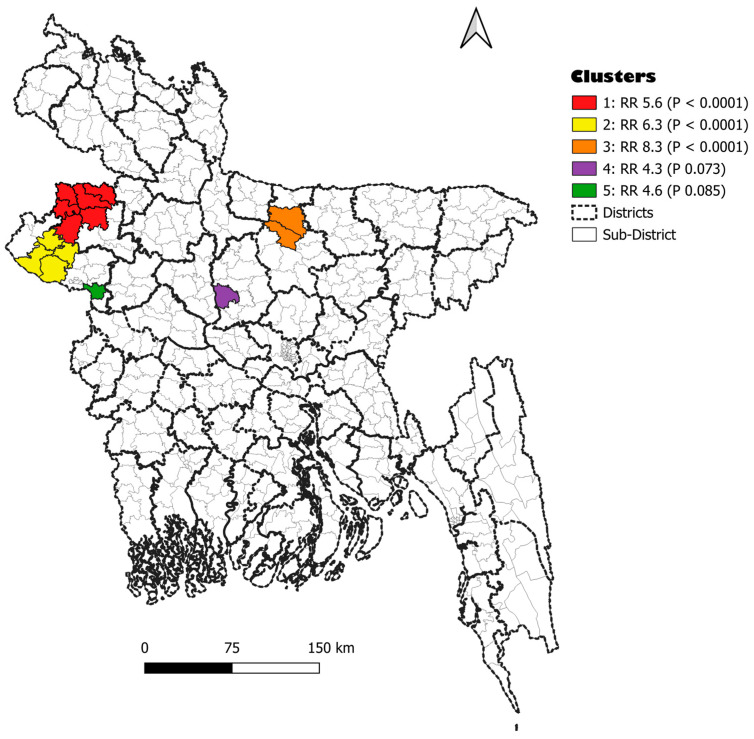
Map of the districts per arm and spatial clusters of new leprosy patients identified at the subdistrict level, Bangladesh, 2020–2023.

A space–time analysis examined new leprosy patients at the subdistrict level, and we found eight high-incidence clusters, of which three were statistically significant and two were borderline significant (*p* < 0.10). Details are shown in Table 3 and Figure 3.

## 4. Discussion

The active screening intervention in this study was feasible, with 1482 household contacts and 25,720 neighborhood contacts screened. This effort identified 7 new cases among household contacts and 18 new cases among neighborhood contacts, findings which were similar to other high endemic settings [18]. Unexpectedly, the proportion of G2D disability was higher (15%) in the intervention compared to the control arm (11%). We hypothesize that long-term active case findings must be implemented in order to achieve a reduction in G2Ds. Moreover, the yield of active case finding among neighborhood contacts was lower than expected, as a result of which most cases included in the study were index cases rather than secondary cases identified as a result of contact screening.

In this study, the proportion of MB among index cases was higher in the intervention arm (56%) than in the control arm (48%). In contrast, among neighbor contacts a high proportion of PB (89%) was detected. In the intervention arm, the proportion of children among new cases detected was 11% in neighbor contacts, superior to the nationwide average (6%). We therefore need to sustain ACF until no children are detected among new cases in five years to stop the transmission of *M. leprae*.

The spatial cluster analysis found three statistically significant clusters during the years 2022–2023, after the years of the COVID-19 restriction measures implemented between 2020 and 2022. The first cluster showed a 5.6-fold higher relative risk (RR) of leprosy incidence in subdistricts included in the cluster compared to subdistricts not included in the cluster. The second cluster had nearly a 6.3-fold increase in RR, and the third cluster had an 8.3 RR. Targeting spatial clusters could more efficiently reduce transmission. However, we will need to assess clustering at lower administrative areas to be cost-efficient. A methodology to outline hamlets and assess clustering at this level could be applied [11], as most of the districts where the Damien Foundation supports leprosy control are located in rural areas.

This study also identified 18 cases among neighborhood contacts, which supports the importance of extended contact screening, even if the yield was less than expected. Also, our study was heavily affected by the COVID-19 pandemic and therefore the number of people screened per index case may have been relatively low. Including index cases diagnosed in a retrospective period of 5–10 years could improve efficiency.

## 5. Conclusions

Screening door-to-door neighbor contacts as well as household contacts in a buffer of 75 m from an index case was feasible and found additional new cases in neighbor contacts. However, we were not able to show a reduced G2D prevalence among new cases in the intervention areas, which would have been a sign of early case detection. Moreover, the yield of new cases among neighbor contacts was lower than expected. There was spatial clustering of new leprosy patients in the year 2023 coinciding with easing of restrictions on active case detection activities in place during the COVID-19 pandemic, probably indicative of a backlog in case detection.

## Figures and Tables

**Table 1:** Sociodemographic data and clinical characteristics of index cases enrolled in intervention and control arms, Bangladesh, 2020–2023.

Factor	Category	Intervention Arm	Control Arm
		n = 347 (%)	n = 380 (%)
Gender	Male	199 (57)	221 (58)
	Female	148 (43)	159 (42)
Age	<15 years	34 (9.8)	36 (9.5)
	15–90 years	313 (90)	344 (90)
Median Age (years)		40 (29–53) *	40 (26–65) *
Type of Leprosy	Multibacillary	195 (56)	183 (48)
	Paucibacillary	152 (44)	197 (52)
Grade of Disability	0	228 (66)	267 (70)
	1	66 (19)	71 (19)
	2	53 (15)	42 (11)

* Interquartile range.

**Table 2:** Sociodemographic data and clinical characteristics of additional cases detected, Bangladesh, 2020–2023.

Item	Category	Intervention Arm	Control Arm
		N = 25 (%)	N = 11 (%)
Gender	Male	13 (52)	6 (55)
	Female	12 (48)	5 (45)
Age	<15 years	3 (12)	5 (45)
	15–90 years	22 (84)	6 (55)
Type of Leprosy	Multibacillary	4 (16)	2 (18)
	Paucibacillary	21 (84)	9 (82)
Grade of Disability	0	20 (80)	11 (100)
	1	4 (16)	0 (0)
	2	1 (4)	0 (0)

**Table 3:** Space–time clusters of new leprosy patients identified at the subdistrict level, Bangladesh, 2020–2023.

Cluster	Number of Locations	Start Date	End Date	Population	New Cases	RR	***p*-Value**
1	6	1 January 2022	31 December 2023	1,429,599	100	5.6	<0.0001
2	5	1 January 2022	31 December 2023	1,120,238	83	6.3	<0.0001
3	2	1 January 2021	31 December 2022	534,263	52	8.3	<0.0001
4	1	1 January 2021	31 December 2022	269,873	11	4.3	0.073
5	1	1 January 2021	31 December 2022	316,539	10	4.6	0.085

(RR = relative risk).

## Data Availability

The database of this study is stored at the Damien Foundation, Belgium, and will not be made openly accessible because of ethical and privacy concerns. However, data can be shared after approval of a motivated and written request to MTU@damiaanactie.be.
